# Embryonic background risk promotes the survival of tadpoles facing surface predators

**DOI:** 10.1371/journal.pone.0193939

**Published:** 2018-03-21

**Authors:** Adam L. Crane, Douglas P. Chivers, Maud C. O. Ferrari

**Affiliations:** 1 Department of Biology, University of Saskatchewan, Saskatoon, Saskatchewan, Canada; 2 Biomedical Sciences, WCVM, University of Saskatchewan, Saskatoon, Saskatchewan, Canada; Uppsala Universitet, SWEDEN

## Abstract

Exposure to intense predation risk can induce morphological and behavioural phenotypes that prepare prey, often at young ages, for surviving attacks from unknown predators. However, previous studies revealed that this survival advantage depended on the predator species. Here, we used alarm cues from injured conspecifics to simulate a period of high predation risk for embryonic wood frogs, *Lithobates sylvaticus*. Two weeks post-hatching, we tested whether the embryonic risk exposure influenced survival in encounters with two novel predators: (1) a spider (*Dolomedes* sp.) that ambushes prey exclusively on the surface of the water, and (2) the adult predacious diving beetle (*Dytiscus* sp.) which displays underwater sit-and-wait posture and pursuit tactics. Tadpoles exposed to embryonic high-risk survived longer when encountering spiders, whereas background risk had no influence on survival with adult beetles. These findings, coupled with survival studies involving other predator types, indicate that a high-risk environment promotes tadpole survival in future encounters with unknown sit-and-wait predators, but at the cost of increased vulnerability to novel predators capable of active pursuit.

## Introduction

The risk of predation has a pervasive influence the lives of prey animals, potentially leading to long-term changes in neurophysiology, morphology, and behaviour [1,2]. Some species develop thicker armour or longer spines for protection (i.e., induced morphological defences: [3,4], whereas others invest heavily in growth to increase escape performance or to outgrow the gape of predators [5,6]. Inducible behavioural phenotypes are also common, being highly plastic and rapidly acquired and enacted. For example, exposure to predation risk can induce specific behavioural traits, such as hypervigilance, chronic immobility, and neophobia (reviewed in: [7]), which appear particularly long-lasting when acquired at young ages, including during embryonic development (e.g., [8]). Although inducible defences may be too costly to employ when predation risk is low, the increased chances of survival in high-risk environments outweigh those costs [9,10,11]. Often species lack innate recognition of specific predators, and even though they may be capable of learning the identity of predators, surviving the initial encounters with predators remains a challenge. Thus, the acquisition of high-risk phenotypes such as neophobia may be particularly valuable in environments where prey likely face frequent exposure to novel predators [11,12], either those that are novel on an individual level (i.e., no lifetime experience) or those that are evolutionarily novel such as invasive predators [12,13].

Only a few studies have tested whether background risk actually translates into future survival with novel predators. In a study on damselfish, *Pomacentrus chrysurus*, individuals that lacked specific information about their predators were able to survive longer in their natural reef ecosystem after experiencing a high level of background risk [14]. That risk period lasted only 4 d, suggesting a rapid change in behavioural traits such as neophobia, vigilance, or shelter use. Indeed, numerus studies have documented such changes following short periods of background risk [7], whereas morphological changes that are biologically relevant appear unlikely to occur so quickly, but see Relyea [15] for an exception.

Other studies have revealed that whether background risk increases survival in encounters with novel predators can depend on the specific predator species. Benard and Fordyce [16] used repeated exposure to injured conspecific cues (hereafter, alarm cues) to simulate a high-risk environment for predator-naïve tadpoles, *Bufo boreas*. Then, tadpole survivorship was assessed in the presence of native predators. In comparison to low-risk individuals, tadpoles from the high-risk environment showed higher survival in the presence of two predators, dragonfly nymphs (*Aeshna* sp.) and predaceous diving beetle larvae (*Dytiscus* sp.), but not with giant water bugs (*Abedus indentatus*). No significant changes in tadpole morphology were detected, suggesting that increased survival was facilitated by behavioural changes. Dragonfly nymphs stalk their prey, clinging to habitat structure while using a sit-and-wait foraging strategy [17]. Predaceous diving beetle larvae also use habitat structure to spring attacks with their piercing sickle-shaped jaws [18]. Giant water bugs are also piercer-predators but alternate between using a sit-and-wait and active-pursuit tactics [19].

In another study, alarm cues were used to simulate a high-risk environment for wood frog tadpoles, *Lithobates sylvaticus* [20]. Survivorship was increased in the presence of predaceous diving beetles from two genera (*Dytiscus* sp. and *Acilius* sp.), whereas survivorship decreased in the presence of non-native crayfish (*Orconectes virilis*) and trout (*Oncorhynchus mykiss*). Both beetle larvae employed a sit-and-wait foraging strategy, whereas the crayfish and trout were species that actively pursue prey [21,22,23,24]. Taken together with Benard and Fordyce’s [16] study, these findings suggested that a high-risk environment prepares tadpoles for facing the sit-and-wait foraging tactics of many of their native predators. However, a high-risk environment appears to lead to behavioural changes for tadpoles that are maladaptive against novel predators exclusively using an active-pursuit foraging strategy [20].

Our goal here was to test whether embryonic background risk affects survival in novel encounters with a native ambush predator, a fishing spider (*Dolomedes* spp., Clerck 1758), and the adult predaceous diving beetle (*Dytiscus* spp., Linnaeus 1758). The spider predator specializes in ambushing prey exclusively on the surface of the water [25]. Adult predaceous diving beetles forage underwater, but unlike their larval counterparts, little is known about their foraging behaviour. While holding air under their wings, adult beetles dive to locate prey and also scavenge on dead animals. They are engulfer predators, holding and tearing their prey with their mouthparts [19,26]. They often assume postures on habitat structure or just below the surface of the water where their hind legs are positioned forward in preparation of launching a dive (personal observations). Hence, we expected that both spiders and beetles would use sit-and-wait tactics to capture tadpoles in our survival experiments. We predicted that embryonic background risk would promote increased survival for tadpoles facing both predators.

## Materials and methods

### Ethics statement

This study was approved by the University of Saskatchewan’s University Council on Animal Care and Supply (protocol 20060014), being conducted in accordance with guidelines of the Canadian Council on Animal Care. We prepared alarm cues according to amphibian guidelines using standard physical methods [27] rather than chemical methods that could potentially interfere with the chemical nature of the alarm cues [28].

### Species collection and maintenance

We collected 8 clutches of wood frog eggs (within 36 h of oviposition) from ponds in central Alberta, Canada in April 2016. To date, no study has reported innate predator recognition in tadpoles from this field site, so collection as embryos presumably results in predator naïveté. Each clutch was split into two subclutches and housed outdoors in separate 4-l holding containers (~20×20×20 cm) containing water from a well (hereafter, water). The water had been seeded with plankton and aquatic reeds from one of the ponds to provide natural floral odours without predator odours. The holding containers were kept outdoors under canopy cover (>90%) adjacent to one of the ponds.

We collected 13 spiders (9.97 mm snout-vent length ±1.72 SD) by skimming the surface of the pond with a net. We also netted 13 adult beetles (3.25 cm snout-vent length ±0.29 SD) that flew into water troughs at the field site. We housed spiders and beetles in outdoor containers (~20×20×20 cm) with water, aquatic reeds for perching, and a lid to prevent their escape. To standardize hunger levels, spiders and beetles were not fed for 2 days prior to the start of the experiment.

### Background risk

As in previous studies (e.g., [11,20]), we simulated a high-risk environment by repeatedly exposing the eggs to alarm cues. Detection of such cues by nearby conspecifics reliably indicates that a predator attack has occurred (reviewed in aquatic taxa in: [29]). Repeated exposure to alarm cues in the absence of specific information about the identity of the predator is known to cause generalized fear toward novel stimuli (i.e., neophobia) rather than specific predator recognition [11]. To obtain alarm cues, donor tadpoles were rapidly pulverized with a mortar and pestle and the resulting product was filtered to remove any solid particles. Then the liquid was diluted in a small volume of water to reach a concentration of 3 tadpoles (10–12 mm total length) per 10 ml.

One subclutch from each clutch was assigned to a treatment of high risk (exposure to alarm cues), and the other subclutch to low risk (exposure to a water control). We gently injected 20 ml of the cues around the egg mass within the 4-l containers. For the high-risk treatment, this amount represented a high level of threat that is known to elicit a strong behavioral fright response [30,31]. Exposures occurred twice per day (1100–1300 and 1700–1900) until embryos began wriggling just prior to hatching. Upon hatching, tadpoles were fed alfalfa pellets (Wardley) and received a 30% water change every 3 d. Trials occurred when tadpoles were approximately 2 weeks post-hatching, 12–14 mm total length, and at Gosner [32] stage 25.

### Experiment 1: Survival trials with spiders

Survival trials occurred in outdoor holding arenas (~20×20×20 cm), placed in a location at the field site without canopy cover and receiving equal amounts of light and dark. The arenas contained 2 l of water and 1–2 aquatic reeds for predator perching. First, one spider was added to each arena and given ~15 min to acclimate. Then, trials began when either a high-risk or low-risk tadpole was poured from a cup into the centre of each arena. Scan sampling occurred at half-hour intervals to determine the survival time (i.e. latency to consumption) of tadpoles. We evaluated the data when over half of the tadpoles had been consumed, which occurred at 36 h. Hence, we assigned a value of 36 h to spiders that had not consumed the tadpole. Once trials had ended, the spiders were returned to their holding containers with lids, and surviving tadpoles were released in the pond (i.e., not re-tested). After a 2-day intermission, we retested each spider in the same manner except the risk treatment was reversed. Thus, each spider had the opportunity to consume a high-risk tadpole and a low-risk tadpole in a random order. When the experiments were completed, spiders and surviving tadpoles were returned to the ponds.

### Experiment 2: Survival trials with adult beetles

Overall, beetles consumed tadpoles faster than spiders, and survival trials with beetles were evaluated after 18 h. All other details for trials with beetles matched those with spiders. However, during our scan sampling, beetles appeared to show high variability in their foraging behaviour, actively pursuing tadpoles between bouts of ambush posture. This led us to conduct additional observations.

### Experiment 3: Activity assay with adult beetles

After a 2-d intermission, each beetle was placed into the testing arena and given a 15-min acclimation period. Then, one low-risk tadpole was added to each arena, and a 10-min observation period began. We recorded the number of times each beetle spent in active pursuit and the number of times the beetle undertook a new ambush posture.

### Statistical analyses

We used nonparametric tests because our data failed parametric assumptions. In each experiment, we compared the proportion of surviving individuals between the high- and low-risk treatments using McNemar’s tests for related frequencies [33]. We also used Wilcoxon tests to assess differences in the change in survival time between the treatments (i.e., the time for high risk—the low risk), where each individual predator served as its own control [33]. To assess the potential influence of predator size, we used Spearman Rank tests for each treatment, adjusting the experimental-wise error rate with Bonferroni corrections (α/2 = 0.025). For survival trials with beetles, we also explored potential correlations between the survival time of high- and low-risk tadpoles and the behaviour of beetles in our activity assay, again using Spearman Rank tests with Bonferroni corrections.

## Results

### Experiment 1: Survival trials with spiders

**Fig 1:**
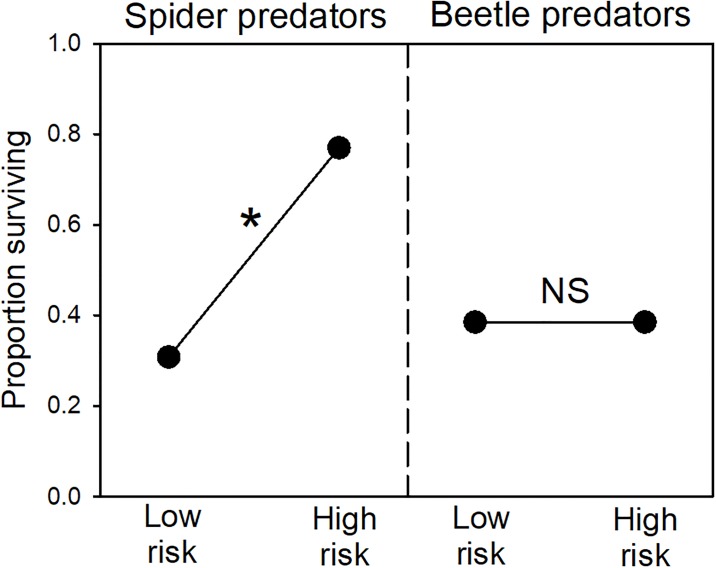
Tadpole survival rate. The proportion of surviving low- and high-risk tadpoles in experiments with spider and beetle predators (*n* = 13 per group). The asterisk represents statistical significance (NS = nonsignificant).

**Fig 2:**
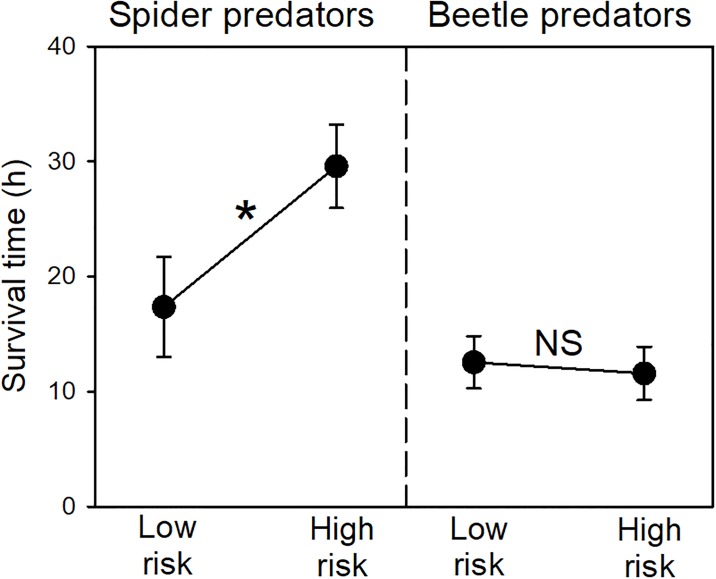
Tadpole survival time. Mean (±SE) change in survival time for high risk tadpoles (compared to low) in experiments with spider or beetle predators (*n* = 13 per group). The asterisk represents statistical significance (NS = nonsignificant).

**Fig 3:**
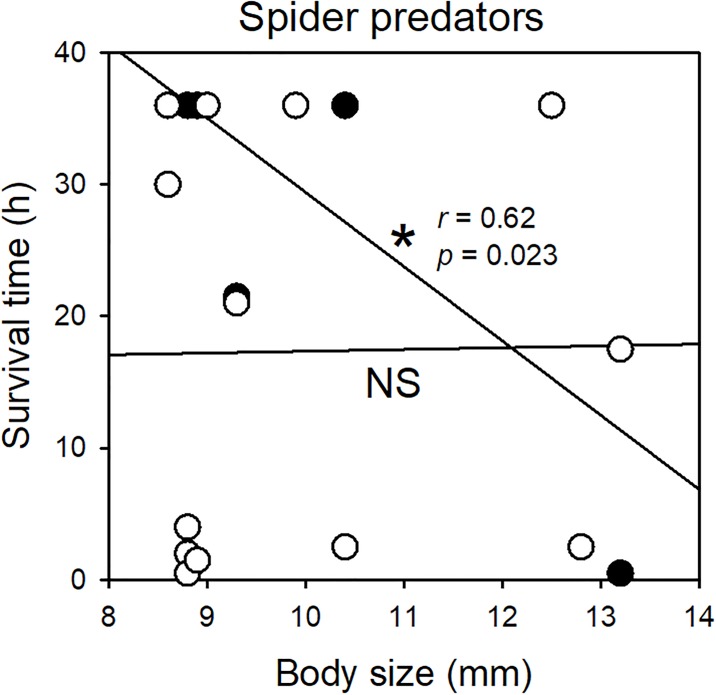
Predator size relationships. Tadpole survival times for low-risk (open circles) and high-risk (closed circles) across predator body size for spider predators (*n* = 13 per group). The asterisk represents statistical significance (NS = nonsignificant).

After the 36-h trials with spiders, significantly more high-risk tadpoles had survived, compared to low-risk individuals (*χ*^*2*^_*1*_ = 5, *P* = 0.025; Fig 1). This difference in survivorship resulted in nearly a doubling of survival time for high-risk tadpoles (*Z* = 2.1, *P* = 0.036; Fig 2). Predator size showed no relationship with survival time for low-risk tadpoles (*r* = 0.06, *P* = 0.85). However, there was a significant negative relationship for high-risk tadpoles, where survival time was shorter when spiders were larger (*r* = 0.62, *P* = 0.023; Fig 3).

### Experiment 2: Survival trials with adult beetles

In 18-h trials with beetles, we found no differences in survival of high- and low-risk tadpoles. Survival rate was similar (*χ*^*2*^_*1*_ < 0.01, *P* > 0.50; Fig 1), as was the survival time (*Z* = 0.08, *P* > 0.50; Fig 2). Moreover, there were no correlations between predator size and survival time for either treatment (low risk: *r* = 0.24, *P* = 0.42; high-risk: *r* = 0.26, *P* = 0.40; not depicted).

### Experiment 3: Activity assay with adult beetles

In our assays of beetle activity, three beetles were successful in consuming the tadpole during the 10-min period (handling time ranged from 1–60 s). Because these beetles changed their behaviour upon subjugation, they were omitted from further analysis, but surprisingly, each of the successful beetles captured the tadpole by active pursuit. On average, beetles spent a little over half their time in pursuit of tadpoles [5.47 (±2.89 SD) min], while also displaying 3.3 (±2.9 SD) discrete bouts of ambush posture. However, neither variable correlated with capture times during the survival trials (time in pursuit: high-risk *r* = 0.34, *P* = 0.25, low-risk *r* = 0.15, *P* = 0.62; bouts of ambush posture: high-risk *r* = 0.11, *P* = 0.73, low-risk *r* = 0.25, *P* = 0.41; not depicted).

## Discussion

We found that short term exposure to risk during the embryonic period had a marked influence on tadpole survival. Moreover, the influence of background risk depended on which predator species the tadpole encountered. Risk promoted increased survival during encounters with spiders but had no effect on survival in encounters with adult beetles. To our knowledge, this is the first study to assess tadpole survival with either of these predators, adding to the growing body of literature on how young amphibians manage predation risk from a diversity of novel native predators.

Differences in survival during encounters with spiders may have resulted from changes in both behaviour and morphology [34]. Tadpoles had about two weeks to develop morphological changes after their embryonic exposure to risk. In another study, we found that wood frog tadpoles exposed to embryonic background risk increased tail depth and decreased activity (unpublished data). A deeper tail enhances escape responses, whereas reduced activity presumably serves to limit their detection by predators while tadpoles still have relatively weak locomotor ability [35,36]. While we did not record behaviour of tadpoles during trials, high-risk individuals appeared to spend more time on the bottom of the arenas, whereas low-risk individuals would swim to the surface more often, allowing the sit-and-wait spiders to make successful strikes. Because any changes in tail depth are unlikely to influence the frequency of surfacing behaviour in tadpoles, we presume that induced behavioural changes were the primary driver of survival differences in our experiment with spiders. We also found that the survival time of high-risk tadpoles decreased with increasing spider size, indicating that the larger ambush radius of larger spiders allowed them to take advantage of the rare surfacing behaviour of high-risk individuals. Another possibility is that the larger spiders were simply more aggressive overall, although previous work with *Dolomedes* sp. has not supported such a pattern [37].

Reduced activity levels also lower the probability of moving near an underwater sit-and-wait predator, promoting survival with beetle larvae and dragonfly nymphs, as documented in previous studies [16,20]. However, contrary to our expectation that adult beetles would mainly employ sit-and-wait tactics, our activity assays revealed that beetles spent about half of their time in active pursuit. This pattern was primarily driven by individuals using both strategies; in fact, only two of 13 beetles spent under 30% of their time in active pursuit. This mix of strategies may explain why neither high- or low-risk tadpoles had a survival advantage with beetles and why neither strategy was more successful for capturing tadpoles from the high- or low-risk background. In Benard and Fordyce’s [16] study, a similar outcome was observed with giant water bug predators that use both sit-and-wait and active-pursuit strategies.

At our site, wood frog tadpoles face a diversity of predators not limited to diving beetles and spiders, but also including dragonfly nymphs (e.g., *Aeshna* spp.), birds, and the occasional tiger salamander, *Ambystoma mavortium*. Of these, the voracious larvae of the predacious diving beetle far outnumber other types of predators, while none appear to exclusively use an active-pursuit foraging strategy (personal observations). Adult diving beetles can lay several eggs during their annual oviposition period (e.g., n = ~20 in: [38]), making larvae far more common than adults initially, although we have little information on predator densities in natural ponds, or on the abundance of the larval diving beetles compared to adults [39].

From an evolutionary perspective, exposure to high-risk appears to induce behaviour (reduced activity) that prepares naïve wood frog tadpoles for encounters with their abundant sit-and-wait predators [20,40,41]. At the same time, reducing activity makes these tadpoles easier targets for novel predators capable of active-pursuit such as adult beetles, or introduced predators [20]. This foraging advantage for active-pursuit predators could potentially be maintained due the dominant predation pressure from sit-and-wait predators. If active-pursuit predators are rare, tadpoles may adopt an antipredator rule of initially responding to risk in a manner that facilitates avoidance of sit-and-wait tactics even when those are not the tactics being used. How learning and inducible changes in body size by prey might negate this initial advantage for active-pursuit predators, and perhaps at the cost of becoming more vulnerable to sit-and-wait predators, is a topic of further interest. We know that wood frog tadpoles are capable of quickly learning the identity of predators [42], as well as changes in the intensity of predation risk [31], but learning to respond correctly to rare or novel predator tactics is presumably more difficult, as some research suggests [43].

## Supporting information

S1 File Data. All data for this manuscript are available as electronic supporting information.
(XLSX)
